# Tracking Changes in Mobility Before and After the First SARS-CoV-2 Vaccination Using Global Positioning System Data in England and Wales (Virus Watch): Prospective Observational Community Cohort Study

**DOI:** 10.2196/38072

**Published:** 2023-03-08

**Authors:** Vincent Nguyen, Yunzhe Liu, Richard Mumford, Benjamin Flanagan, Parth Patel, Isobel Braithwaite, Madhumita Shrotri, Thomas Byrne, Sarah Beale, Anna Aryee, Wing Lam Erica Fong, Ellen Fragaszy, Cyril Geismar, Annalan M D Navaratnam, Pia Hardelid, Jana Kovar, Addy Pope, Tao Cheng, Andrew Hayward, Robert Aldridge, Virus Watch Collaborative

**Affiliations:** 1 Centre for Public Health Data Science Institute of Health Informatics University College London London United Kingdom; 2 Institute of Epidemiology and Health Care University College London London United Kingdom; 3 SpaceTimeLab Department of Civil, Environmental and Geomatic Engineering University College London London United Kingdom; 4 Technical Research Department Esri Edinburgh United Kingdom; 5 Department of Infectious Disease Epidemiology London School of Hygiene and Tropical Medicine London United Kingdom; 6 Department of Population, Policy and Practice University College London Great Ormond Street Institute of Child Health London United Kingdom; 7 See Acknowledgements

**Keywords:** COVID-19, SARS-CoV-2, vaccination, global positioning system, GPS, movement tracking, geographical tracking, mobile app, health application, surveillance, public health, mHealth, mobile surveillance, tracking device, geolocation

## Abstract

**Background:**

Evidence suggests that individuals may change adherence to public health policies aimed at reducing the contact, transmission, and spread of the SARS-CoV-2 virus after they receive their first SARS-CoV-2 vaccination when they are not fully vaccinated.

**Objective:**

We aimed to estimate changes in median daily travel distance of our cohort from their registered addresses before and after receiving a SARS-CoV-2 vaccine.

**Methods:**

Participants were recruited into Virus Watch starting in June 2020. Weekly surveys were sent out to participants, and vaccination status was collected from January 2021 onward. Between September 2020 and February 2021, we invited 13,120 adult Virus Watch participants to contribute toward our tracker subcohort, which uses the GPS via a smartphone app to collect data on movement. We used segmented linear regression to estimate the median daily travel distance before and after the first self-reported SARS-CoV-2 vaccine dose.

**Results:**

We analyzed the daily travel distance of 249 vaccinated adults. From 157 days prior to vaccination until the day before vaccination, the median daily travel distance was 9.05 (IQR 8.06-10.09) km. From the day of vaccination to 105 days after vaccination, the median daily travel distance was 10.08 (IQR 8.60-12.42) km. From 157 days prior to vaccination until the vaccination date, there was a daily median decrease in mobility of 40.09 m (95% CI –50.08 to –31.10; *P*<.001). After vaccination, there was a median daily increase in movement of 60.60 m (95% CI 20.90-100; *P*<.001). Restricting the analysis to the third national lockdown (January 4, 2021, to April 5, 2021), we found a median daily movement increase of 18.30 m (95% CI –19.20 to 55.80; *P*=.57) in the 30 days prior to vaccination and a median daily movement increase of 9.36 m (95% CI 38.6-149.00; *P*=.69) in the 30 days after vaccination.

**Conclusions:**

Our study demonstrates the feasibility of collecting high-volume geolocation data as part of research projects and the utility of these data for understanding public health issues. Our various analyses produced results that ranged from no change in movement after vaccination (during the third national lock down) to an increase in movement after vaccination (considering all periods, up to 105 days after vaccination), suggesting that, among Virus Watch participants, any changes in movement distances after vaccination are small. Our findings may be attributable to public health measures in place at the time such as movement restrictions and home working that applied to the Virus Watch cohort participants during the study period.

## Introduction

**Figure 1:**
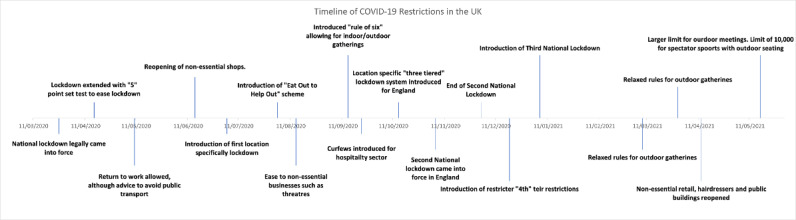
Timeline of restriction events in the United Kingdom during the COVID-19 pandemic from March 2020 to May 2021 (adapted from the Institute for Government [6], which is published under Creative Commons Attribution 4.0 International License [CC BY-NC]).

The UK response to the COVID-19 pandemic has included multiple rounds of restrictions on nonessential movement to reduce contacts and control transmission [1]. Examples of permissible travel included necessary shopping, exercise, medical need, or travel to and from essential work [2]. However, the restriction of movement can have a detrimental impact on a wide variety of outcomes such as reduced physical activity [3], mental health, domestic accidents, the economy, and education [4]. Adherence to travel-based public health interventions and those especially aimed at limiting nonhousehold contact has varied through time [5]. A brief timeline of the United Kingdom’s approach to restrictions can be found in Figure 1 [6].

The introduction of vaccinations against COVID-19 reduces SARS-CoV-2 transmission and disease [7] and, as a result, is a critical part of the strategies to allow more normal societal mixing due to increased immunization. However, in the UK context, there are concerns that misunderstandings about the effectiveness of the COVID-19 vaccine after the first dose, which may be leading to a reduction in adherence to other public health policies and increased exposure of partially protected individuals [8]. Preliminary research on vaccination in February 2021 found that 41% of individuals age >80 years who had their first dose of the vaccine had met at least one other person outside their household within 3 weeks. These contacts did not include care workers or members of their support bubble that were permitted by the restrictions in place at the time [8]. This finding is concerning as antibody levels would not have risen in the 1-2 weeks following the first dose of vaccine [9,10]. Further evidence also suggests that those aged >80 years are more likely to have a positive polymerase chain reaction test in the first 9 days after vaccination compared to a control group, which might be explained by increased mobility and contacts between people in the period following vaccination [11]. During the emergence of the Delta (B.1.617.2) SARS-CoV-2 variant, the effectiveness of both the Oxford-AstraZeneca and Pfizer-BioNTech vaccines were estimated to be 33% against symptomatic disease after a first dose [12], although protection against hospitalization appears to be much higher [13]. Therefore, if those who are not fully vaccinated increase their level of social contact and mobility after vaccination, their risk of becoming infected and infecting others may also be increased.

Understanding movement after the first vaccination is important, as it could help policy makers understand how perceived protection from the vaccination program may negatively offset the effectiveness of other policies designed to reduce transmission. Such arguments can also be extended beyond the first vaccination with the introduction of booster shots and variant-specific vaccines in relation to future SARS-CoV-2 variants. Although previous studies have attempted to investigate travel distances after vaccination [14], these studies were conducted using mobile call data based on cellular tower location, which is considered less accurate compared to GPS location. Alternative methods for movement tracking exist, such as tracking debit/credit card usage [15] and QR code check-in for venues [16]. Although such technology can be used to track specific activities, it can be limited as they require active interaction from users, and assessing travel distance can be limited if travel occurs beyond the use of such technology. GPS technology, on the other hand, uses satellites to allow users to passively submit longitude and latitude data globally, both indoors and outdoors [17].

In this analysis, we aimed to quantify the effect that the first SARS-CoV-2 vaccination had on travelling behavior, using mobile phone GPS data collected from study participants who consented and voluntarily downloaded the ArcGIS Tracker app onto their mobile phones.

## Methods

### Study Design and Setting

The study design used prospective observational data from the Virus Watch cohort; a full description of the Virus Watch study has been published previously [18]. Briefly, households were recruited starting in mid-June 2020, which was aimed at creating a representative cohort of England and Wales. To rapidly recruit participants at the start of the pandemic, we used a range of methods aimed at creating a representative cohort of England and Wales. We used the Royal Mail Post Office Address File to generate a random list of residential addresses that were sent recruitment postcards (n=3914), placed social media advertisements on Facebook and Twitter (n=18,594), and sent SMS text messages (n=11,151) and letters to participants and households from their general practitioners (n=3803). We invited a random sample of eligible participants to our tracker subcohort who were adults (aged ≥18 years) on entry that agreed to participate in installing the ArcGIS Tracker on their compatible smartphone and who provided full details on gender, ethnicity, and a registered address.

**Figure 2:**
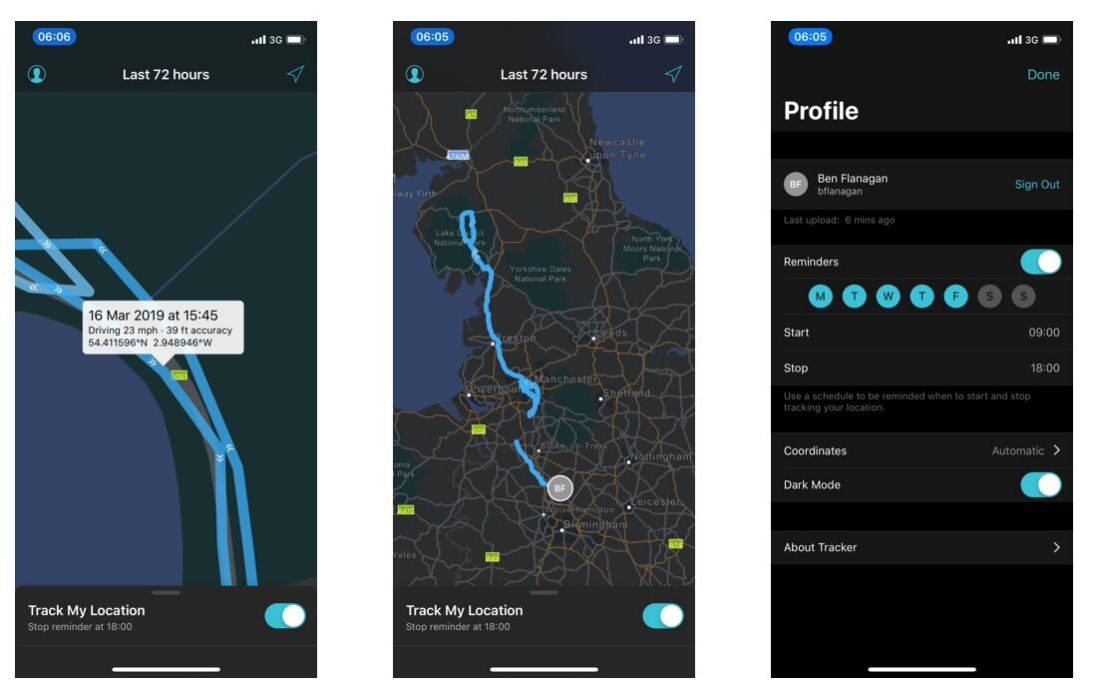
Screenshots of the ArcGIS Tracker app. Esri UK [20], Esri [21], HERE [22], Garmin [23], FAO [24], NOAA [25], USGS [26] are data providers for Esri basemaps.

The ArcGIS Tracker app is available for the Apple iOS (iOS 12 or later) and Google Android (version 5.0 or later) platforms and can be downloaded from their respective app stores [19]. The ArcGIS Tracker app requires users to log in (using provided credentials) and share their location using an on-off toggle button that allows users to control when and where they would like to share their location. The location app is designed to run in the background to collect data; however, on certain models of phones, this function had to be enabled in the smartphones’ settings menu. Using the ArcGIS Tracker app, the subcohort provided the following data: date; time; longitude; latitude; travel mode; and GPS accuracy, which was defined by the phone manufacturer’s GPS algorithm. See Figure 2 for screenshots of the ArcGIS Tracker app.

### Intervention

The date of receiving the first dose of vaccine was self-reported through the weekly Virus Watch questionnaire. We began collecting weekly vaccination status on January 11, 2021, and asked about any prior vaccination during the first 2 weekly surveys. Subsequently, participants were asked to provide a weekly update only. The options available were “Pfizer/BioNTech,” “Oxford/AstraZeneca,” “Moderna,” and “Other/Can’t remember.”

### Study Population

The study population included adults (aged ≥18 years on entry) in the subcohort of the Virus Watch study who were vaccinated and submitted at least 10 days of readings. Participants had to submit at least 5 days of readings before and after their self-reported vaccination date. We excluded readings that were outside of England and excluded analysis from days where there were fewer than 5 contributors. We only used location readings with an accuracy rating of less than 30 m.

### Study Period

We started sending out invitations for the Tracker cohort between September 2020 and February 2021, with the data extraction for this analysis being undertaken in May 2021.

### Outcomes

The unit of the analysis was the aggregated median daily travel distance, with the outcome being the change in median daily travel distance. To calculate the group’s median daily travel distance, we aggregated the daily movement from each participant’s registered address for each day. The median daily distance was chosen to account for the distribution of the cohort’s daily travel patterns.

### Analysis

For each individual and each day, we calculated the cumulative outdoor travel distance recorded using the ArcGIS Tracker app from their registered household address. This was calculated by summing up the distance (*d*) computed by the Euclidean distance method (equation 1) between the 2 sequential outdoor GPS records. Considering the accuracy of the GPS records, we set up a 25 m radius buffer zone (the average horizontal accuracy is 25 m) around a participant’s home location. Points that fall within the buffer are considered as at-home travel activities and therefore considered as zero distance in analyses.

The equation to calculate the distance travelled by each participant is as follows:



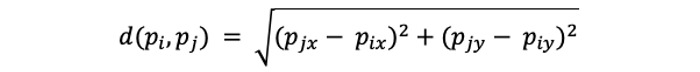



where *d*(*p_i_*, *p_j_*) is the Euclidean distance between 2 sequential GPS points (ie, *p_i_* and *p_j_*); the Cartesian coordinates are (*p_ix_*, *p_iy_*) for *p_i_* and (*p_jx_*, *p_jy_*) for *p_j_*. We used the British National Grid as the reference system.

Our statistical analysis was conducted using an interrupted time series, where we used segmented linear regression to estimate the trends in travel patterns, with the first segment estimating the median travel distance for the cohort before vaccination and the second segment estimating the median travel distance for the cohort after vaccination. Therefore, we defined the interruption time point in our analysis as the date of the first vaccination for each individual, with negative days denoting days prior to vaccination and positive days denoting days after vaccination; for each day, we then calculated the median travel distance.

To calculate the travel trajectory before vaccination, we conducted linear regression analysis using data before vaccination to estimate the sample’s median daily travel distance from their home with time (days before vaccination) as the explanatory variable. To calculate the travel trajectory after vaccination, we conducted linear regression analysis using data after vaccination to estimate the sample’s median daily travel distance from their home with time (days after vaccination) as the explanatory variable. For both models, each day represented 1 data point, with the points for each day being the median travel distance of those who submitted readings on that day. The segmented regression equations can be found in equation 2; linear regression was chosen a priori as we expected the limitations on movement to create a stable pattern in movement. Our alternative hypothesis was that after vaccination, we would see an increase in movement that would be expressed if *a*_2_ > *a*_1_ (a slope change) or *b*_2_ > *b*_1_ when *a*_2_ ≥ *a*_1_ (a level change) [27] in equation 2.

Equation 2 uses segmented linear regression models with model (and subscript) 1 representing the trends before vaccination and model (and subscript) 2 representing the trends after vaccination; *y_n_* represents the estimated median daily travel distance with coefficient *a_n_*, *x* represents the days since vaccination (negative for model 1 and positive for model 2), and *b_n_* is a constant:

*y*_1_ = *a*_1_*x* + *b*_1_ for *x* < 0 **(2)**

*y*_2_ = *a*_2_*x* + *b*_2_ for *x* > 0

The UK vaccination program prioritized people by (older) age and clinical risk groups, which, in addition to differences in the socioeconomic backgrounds between those invited and accepting a vaccination, meant that selecting an appropriate control group for this analysis was not feasible.

### Covariates

Due to the study design, which compared the same individuals’ movement before and after vaccination, we did not use covariates for regression adjustment. For each eligible individual, we used the following data: days since vaccination and the total travel distance for the corresponding day.

### Sensitivity Analyses

We performed various sensitivity analyses. First, after reviewing the data, we repeated the analyses with outliers removed. Three cutoff points were used: days with a median travel distance lower than 50 km, days with a median travel distance lower than 25 km, and days with a median travel distance lower than the median travel distance on the day of vaccination. This third cutoff point was used as the day of vaccination is the 1 day in which we were sure people had travelled (to be vaccinated).

Our second sensitivity analysis accounts for the effect of the removal of national restrictions on movement as alternative explanations for differences in movement after vaccination. We conducted a sensitivity analysis that limited travel and vaccination events to the third national lockdown. This period was from January 4, 2021, to April 5, 2021, and represents a time period when restrictions did not change in relation to rules regarding travel and social distancing.

### Ethics Approval

The Virus Watch study was approved by the Hampstead National Health Service Health Research Authority Ethics Committee (20/HRA/2320). All members of participating households provided informed consent for themselves and, where relevant, for children that they were responsible for. To contribute to the tracker subcohort of Virus Watch, adults had to provide explicit consent during our registration process.

### Information Governance

This research was registered with the University College London (UCL) data protection office and reviewed by the UCL information security and governance teams. The Virus Watch Data Privacy Impact Assessment can be found on the web [28]. During the consent and registration process, adult participants were invited to contribute geolocation data using the ArcGIS mobile phone tracker app. For those who chose to participate, we sent personal identifiable data from the UCL data safe haven to a secure memory stick on a UCL computer, from which we transferred the data via HTTPS into the ArcGIS Online (Esri UK) subscription. The purpose of this data transfer was to set up participants’ tracker app accounts. The transferred data, along with the account passwords, were stored in North America. The UCL Virus Watch study team undertook the data transfer process and had access to the Participant Profile within the ArcGIS Online subscription. Only a small number of named ArcGIS employees had access to the participant profile area and only for the purposes of assisting the UCL study team when necessary. Once the tracker app accounts were created, the UCL study team emailed tracker app participants instructions on how to download the app and sign into the tracker app.

The geolocation data collected by the app were stored securely on a section of the ArcGIS Online subscription hosted in Europe, which is securely cleared every 30 days. Participants’ geolocation data were transferred on a regular basis via HTTPS to a secure memory stick on a UCL machine and were then imported via a secure gateway technology onto the UCL secure memory stick and into the UCL data safe haven.

Geolocation data were linked with other participant study data in the data safe haven. Once analyzed, aggregate data (generated from geolocation and other study data) were exported from the data safe haven and published on the public study website and in research publications.

The aggregated data set used in this analysis will be securely destroyed after 20 years, in line with UCL’s record retention policy. In line with policies developed for electronic health care research, we did not report any data with a cell containing <5 events, and where necessary, we protected these counts with secondary suppression.

## Results

### Cohort Demographics

**Figure 3:**
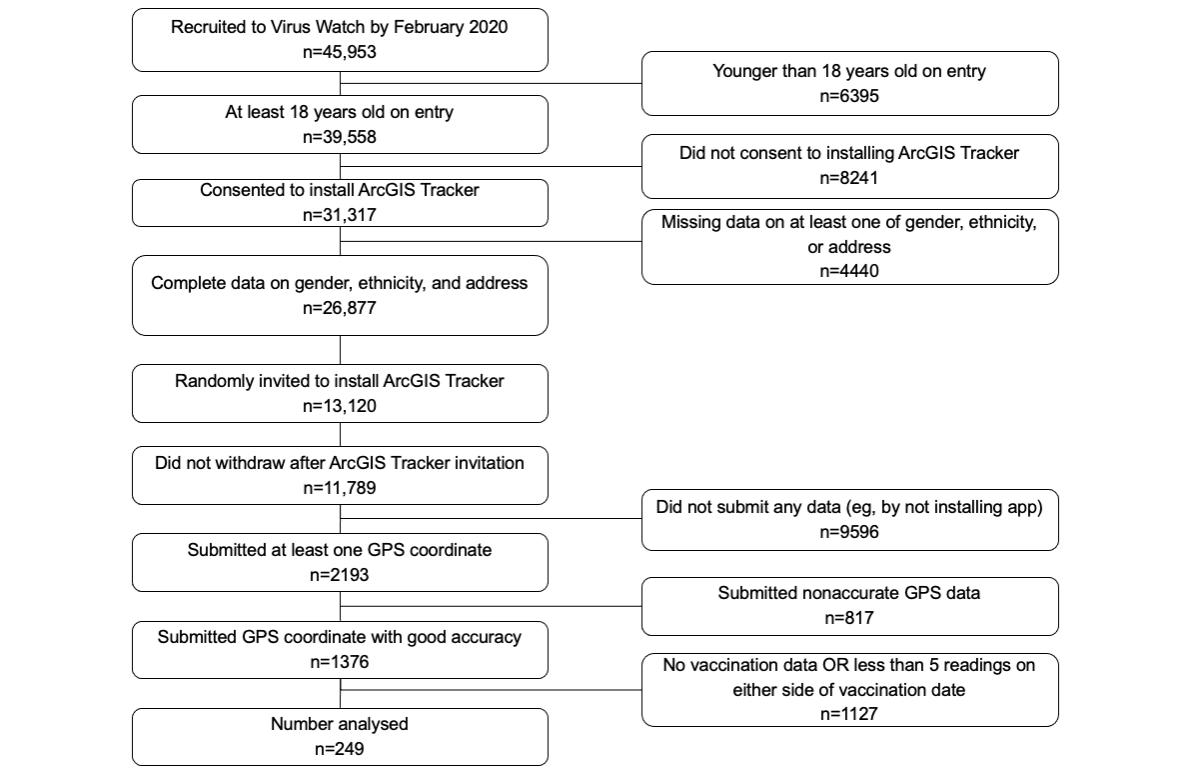
Consolidated Standards of Reporting Trials (CONSORT) diagram for how the cohort was derived.

By February 2021, Virus Watch recruited a total of 45,963 individuals, of whom 39,558 were at least 18 years old when registering. Of these 39,558 individuals, 79% (n=31,317) provided consent to install the ArcGIS Tracker app. Of the 31,317 participants, a sample of 13,120 adults (aged ≥18 years on entry) were chosen to be randomly invited based on having complete information on gender, ethnicity, and address details. Of these participants, 2193 contributed at least one GPS reading. After removing invalid data points, including those outside of England and those that did not submit accurate readings (eg, points that exhibit extremely high horizontal and vertical accuracy), 1376 participants were included. Of the 1376 individuals, 1244 individuals were vaccinated by May 2021. After removing individuals with fewer than 5 data points on either side of their vaccination date, 249 individuals were included in our final analysis. See Figure 3 for the Consolidated Standards of Reporting (CONSORT) diagram of how the cohort was analyzed.

Of the 249 participants, there were more women (n=141, 56.6%) than men, with a median age of 62 (IQR 55-67) years, which is older than the whole Virus Watch cohort. Individual residents in local super output areas in the 3 least deprived quintiles represented 79.1% (n=197) of the population. In all, 88.8% (n=221) of our cohort self-identified as “White—English/Welsh/Scottish/Northern Irish/British,” which is more than the whole Virus Watch cohort. See Table 1 for a sociodemographic breakdown of the cohort.

From the 249 participants, there were 157 days of eligible readings prior to the first vaccination, that is, 157 days before vaccination was the earliest day where there were at least 5 people who submitted readings; there were 105 days of readings under the same eligibility criteria after vaccination. The median number of people who contributed per day was 89 (IQR 34.75-135.50) people, with a median of 91 (IQR 35.00-132.00) people contributing before vaccination and a median of 88 (IQR 30.50-149.25) people contributing after vaccination. The median number of days contributed by the 249 participants was 87 (IQR 58.00-128.00) days, with a median contribution of 51 (IQR 26-77) days before vaccination and a median contribution of 36 (IQR 19.00-56.00) days after vaccination. Zero participants produced data for every day and 5 participants produced an equal amount of data before and after vaccination. Before vaccination (–157 days to –1 day), the median daily travel distance was 9.05 (IQR 8.06-10.09) km, and after vaccination (+1 day to 105 days), the median daily travel distance was 10.08 (IQR 8.60-12.42) km. The median travel distance on the day of vaccination was 19.1 (IQR 8.75-37.90) km.

**Figure 4:**
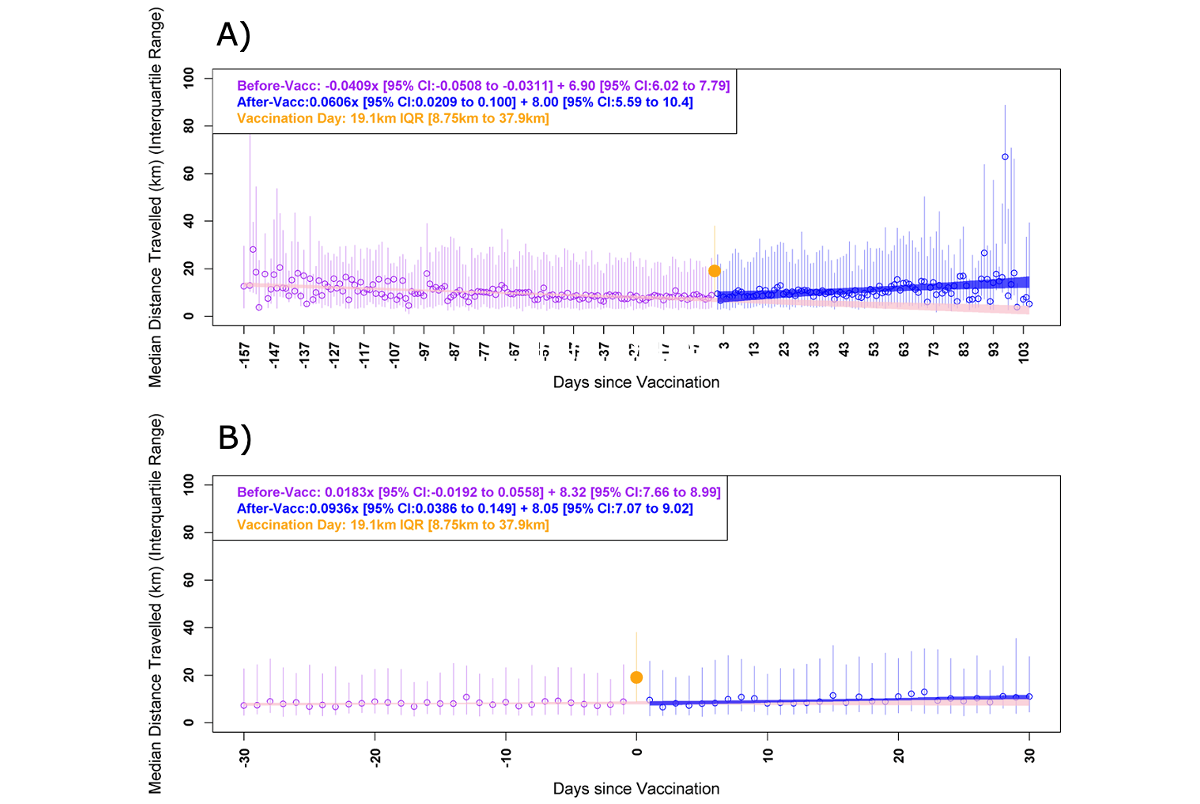
Graphical representation of the interrupted time series. Median daily travel distance from home in reference to vaccination date: (A) unrestricted (primary analysis); (B) only accounting for movement and vaccinations that occurred during the third national lockdown (January 4, 2021 to April 5, 2021) with a 30-day period either side of the vaccination date.

During the first segment of the linear regression model, from 157 days before the first vaccination to the vaccination date, there was a median daily decline of 40.09 m (95% CI –50.08 to –31.10; *P*<.001) of movement with a constant of 6.90 m (95% CI 6.02-7.79; Figure 4A). During the second segment of the linear regression model, from the first vaccination date to 105 days after vaccination, there was a median daily increase of 60.6 m (95% CI 20.9-100; *P*<.001) of movement with a constant of 8.00 m (95% CI 5.59-10.40).

**Table 1:** Sociodemographic breakdown of the included cohort.

Characteristic			Virus Watch cohort by February 2021 (n=45,963)		Invited to tracker cohort and did not withdraw (n=11,789)		Analyzed (n=249)
Age on entry (years), median (IQR)			54 (34-66)		62 (52-69)		62 (55-67)
**Sex, n (%)**							
	Female	21,625 (47)		6589 (55.9)		141 (56.6)	
	Intersex	56 (0.1)		21 (0.2)		0 (0)	
	Male	17,338 (37.7)		5,108 (43.3)		108 (43.4)	
	Missing	6906 (15)		0 (0)		0 (0)	
	Prefer not to say	38 (<0.1)		71 (0.6)		0 (0)	
**Region name, n (%)**							
	East Midlands	3678 (8)		1012 (8.6)		36 (14.5)	
	East of England	9052 (19.7)		2321 (19.7)		50 (20.1)	
	London	6299 (13.7)		1686 (14.3)		40 (16.1)	
	North East	2118 (4.6)		574 (4.9)		5 (2)	
	North West	4598 (10)		1320 (11.2)		23 (9.2)	
	South East	8058 (17.5)		2289 (19.4)		46 (18.5)	
	South West	2992 (6.5)		871 (7.4)		19 (7.6)	
	Wales	1038 (2.3)		306 (2.6)		0 (0)	
	West Midlands	2310 (5)		560 (4.8)		18 (7.2)	
	Yorkshire and the Humber	2049 (4.5)		634 (5.4)		12 (4.8)	
	Unknown	3771 (8.2)		216 (1.8)		0 (0)	
**Index of multiple deprivation quintile, n (%)**							
	1 (most deprived)	4145 (9)		1048 (8.9)		21 (8.4)	
	2	6625 (14.4)		1872 (15.9)		31 (12.4)	
	3	8585 (18.7)		2359 (20)		43 (17.3)	
	4	10,695 (23.3)		2884 (24.5)		74 (29.7)	
	5 (least deprived)	12,142 (26.4)		3410 (28.9)		80 (32.1)	
	Unknown	3771 (8.2)		216 (1.8)		0 (0)	
**Minority ethnicity status, n (%)**							
	Minority ethnicity	5381 (11.7)		1295 (11)		28 (11.2)	
	Missing	7101 (15.4)		0 (0)		0 (0)	
	Prefer not to say	109 (0.2)		84 (0.7)		0 (0)	
	White British	33,372 (72.6)		10,410 (88.3)		221 (88.8)	

### Sensitivity Analysis 1

After reviewing the data, we believe that certain median travel distances were outliers; therefore, we conducted sensitivity analyses to remove days that had readings higher than 50 km, 25 km, and the median distance travelled on the day of vaccination (19.1 km). Under these analyses, all median travel distances before and after vaccination, as well the coefficients and constants, stayed similar (ie, they did not cross the 95% CIs) when compared to the primary analysis. See Table 2 for the coefficients and median travel distances before and after vaccination. See Multimedia Appendices 1-3 for graphical representations of the interrupted time series for these sensitivity analyses.

**Table 2:** Results of various sensitivity analysis involving the removal of outlier points.

Maximum median distance	Median travel distance before vaccination (km; IQR)	Coefficient before vaccination (95% CI)	Constant before vaccination (95% CI)	Median travel distance after vaccination (km; IQR)	Coefficient after vaccination (95% CI)	Constant after vaccination (95% CI)
Unrestricted (primary analysis)	9.05 (8.06 to 11.34)	–0.041 (–0.051 to –0.031)	6.90 (6.02 to 7.79)	10.08 (8.60 to 12.42)	0.061 (0.021 to 0.100)	8.00 (5.59 to 10.40)
50 km	9.07 (8.06 to 11.34)	–0.041 (–0.051 to –0.031)	6.90 (6.02 to 7.79)	10.07 (8.59 to 12.21)	0.034 (0.014 to 0.055)	8.83 (7.63 to 10.00)
25 km	9.05 (8.05 to 11.32)	–0.037 (–0.046 to –0.028)	7.10 (6.29 to 7.90)	10.07 (8.58 to 12.07)	0.028 (0.011 to 0.046)	9.01 (7.95 to 10.10)
19.1 km (vaccination day)	9.05 (8.05 to 11.29)	–0.035 (–0.044 to –0.026)	7.18 (6.40 to 7.97)	10.07 (8.58 to 12.06)	0.029 (0.011 to 0.046)	9.01 (7.95 to 10.10)

### Sensitivity Analysis 2

To account for the effect of national restrictions on movement, we conducted a sensitivity analysis that limited travel and vaccination events to the third national lockdown from January 4, 2021, to April 5, 2021. Due to the asymmetry of the number of data points before and after vaccination in this analysis, we analyzed movement 30 days before vaccination and 30 days after vaccination.

From 30 days prior to vaccination to the vaccination date, there was a median daily movement of 8.06 (IQR 7.49-8.51) km with a median daily movement increase of 18 m (95% CI –19 to 56; *P*=.57). From the vaccination date to the following 30 days (during the third national lockdown), there was a median daily movement of 9.16 (IQR 8.35-10.71) km with a median daily movement increase of 9.35 m (95% CI 39-149; *P*=.69). See Figure 4B for a graphical representation of the interrupted time series for this sensitivity analysis.

## Discussion

### Principal Findings

Our study demonstrates the feasibility of collecting high-volume geolocation data as part of research projects and the utility of these data for understanding public health issues. Our results require cautious interpretation. Our initial analysis found evidence of a modest increase in the rate of change in median daily distance travelled after participants received their first dose of SARS-CoV-2 vaccine, but when restricting our analysis to a period of lockdown, we did not find evidence of a difference in mobility following 1 vaccination dose. On balance, our results do not provide evidence that people increase the rate of their movements following the first vaccination, as the results are consistent with both an increase and no movement after vaccination and suggest that any change in mobility after vaccination is likely to be modest.

We used GPS data to measure the travel distance of vaccinated individuals. Not only does this improve accuracy over other methods of distance estimation by using the GPS system (as compared to cellular location), but it also reduces recall bias when compared with using self-reported data. Our interrupted time series study design aids in reducing the impact of non–time-varying confounders as the same individual’s data are considered before and after vaccination.

### Comparisons With Prior Work

Compared to prior work [14], our methodology builds on that work by using techniques that produce more accurate measures of distance by using the GPS system as compared to cell phone towers. That study took a different-in-difference approach with 1 week of data on either side of vaccination and found that there was an increase in movement after vaccination by 8.6% 1 week after vaccination when compared to the week prior to vaccination. Although our sensitivity analysis, where we restricted to 30 days before and after vaccination, found a 13.6% increase in median movement after vaccination, the CIs of our regression models did not support an overall change in movement and are reflective of the overlapping IQR ranges before (median 8.06, IQR 7.49-8.51 km) and after (median 9.16, IQR 8.35-10.71 km) vaccination.

Given that previous studies have suggested people increase their nonhousehold contacts after their first vaccination, further research on behavior change following vaccination is warranted. In the meantime, it is important that public health communications are clear about the differential protection against SARS-CoV-2 infection offered by the first and second doses of the vaccine, such that people can exercise sound personal judgement on how they alter their behavior following vaccination.

### Limitations

Our studies’ sample size means that we may be underpowered to detect small changes in mobility, particularly when restricting to a period of national lockdown. Our GPS collection was automated but could be switched on and off by participants and is more likely to have been switched off on days when participants stayed at home. The use of the app in this way would result in our analysis overestimating the median distance travelled per day, through the nonreporting of GPS data (eg, switching the app off) on days when participants stayed at home. Due to technological requirements of tracking apps, the results were skewed toward those who had access to a smartphone and were able to contribute their data plan toward research activities, leading to a low initial uptake rate. Furthermore, for those who did provide data, there was an inconsistent provision of data over time, which could be caused by participants switching off the app. With a draining effect on battery life from the GPS app used, the dropout rate from the tracker cohort of Virus Watch was relatively high. People taking part in Virus Watch are self-selecting and motivated to contribute to COVID-19 research, and therefore, their movement patterns may not be generalizable to all vaccinated groups.

An important limitation in our primary analysis is that it does not control for time-varying confounders such as changes in national physical distance rules that were likely to have led to an increase in the rate of change in median daily travel distance. Other time-varying SARS-CoV-2–related events that we could not control for included changes in infection rates, which may have influenced people’s decision to travel. As we centered our analysis around the vaccination date, our analysis did not account for different time-varying public health interventions on different vaccination dates. It is also possible that other non–SARS-CoV-2 events such as weather changes may have affected the findings, with participants increasing mobility during this same time period after vaccination as a result of improvements in weather conditions.

### Conclusions

Although previous research found that nearly half of those who were vaccinated (after 1 dose) met with others outside their households or support bubbles [8], our findings provide a mixed picture about movement after the first dose of vaccination, and we found no evidence of an increase in movement when we conducted our analyses during a period of national movement restrictions.

## Appendix

Multimedia Appendix 1 Sensitivity Analysis 1: graphical representation of the interrupted time series once we removed days that had readings higher than 50 km.

Multimedia Appendix 2 Sensitivity Analysis 1: graphical representation of the interrupted time series once we removed days that had readings higher than 25 km.

Multimedia Appendix 3 Sensitivity Analysis 1: graphical representation of the interrupted time series once we removed days that had readings higher than the median distance travelled on the day of vaccination (19.1 km).

## Abbreviations

CONSORT: Consolidated Standards of Reporting
UCL: University College London
