# Supplementary figures and images for: Tracking Changes in Mobility Before and After the First SARS-CoV-2 Vaccination Using Global Positioning System Data in England and Wales (Virus Watch): Prospective Observational Community Cohort Study
Source: JMIR Public Health Surveill. 2023 Mar 8;9:e38072. doi: 10.2196/38072 (PMC9997704; doi:10.2196/38072)

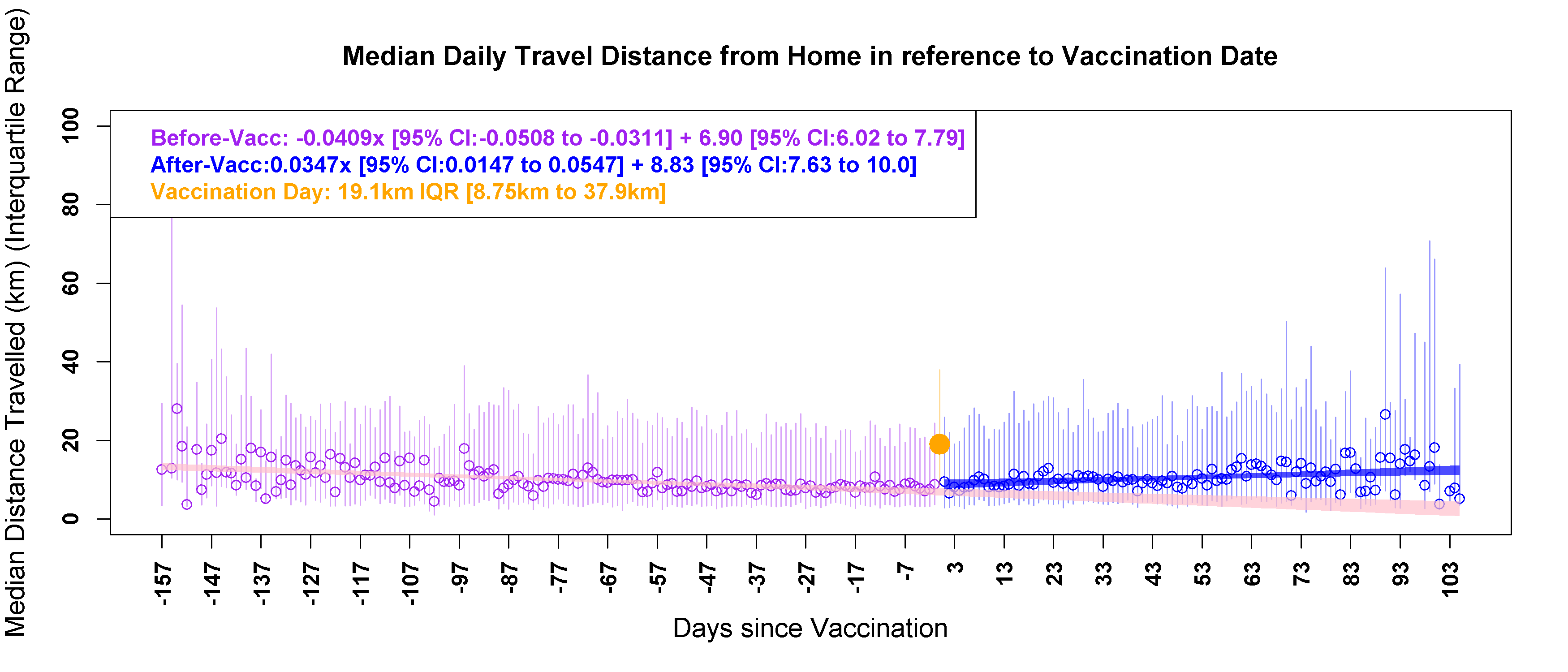

Supplement: Multimedia Appendix 1 [file publichealth_v9i1e38072_app1.png]

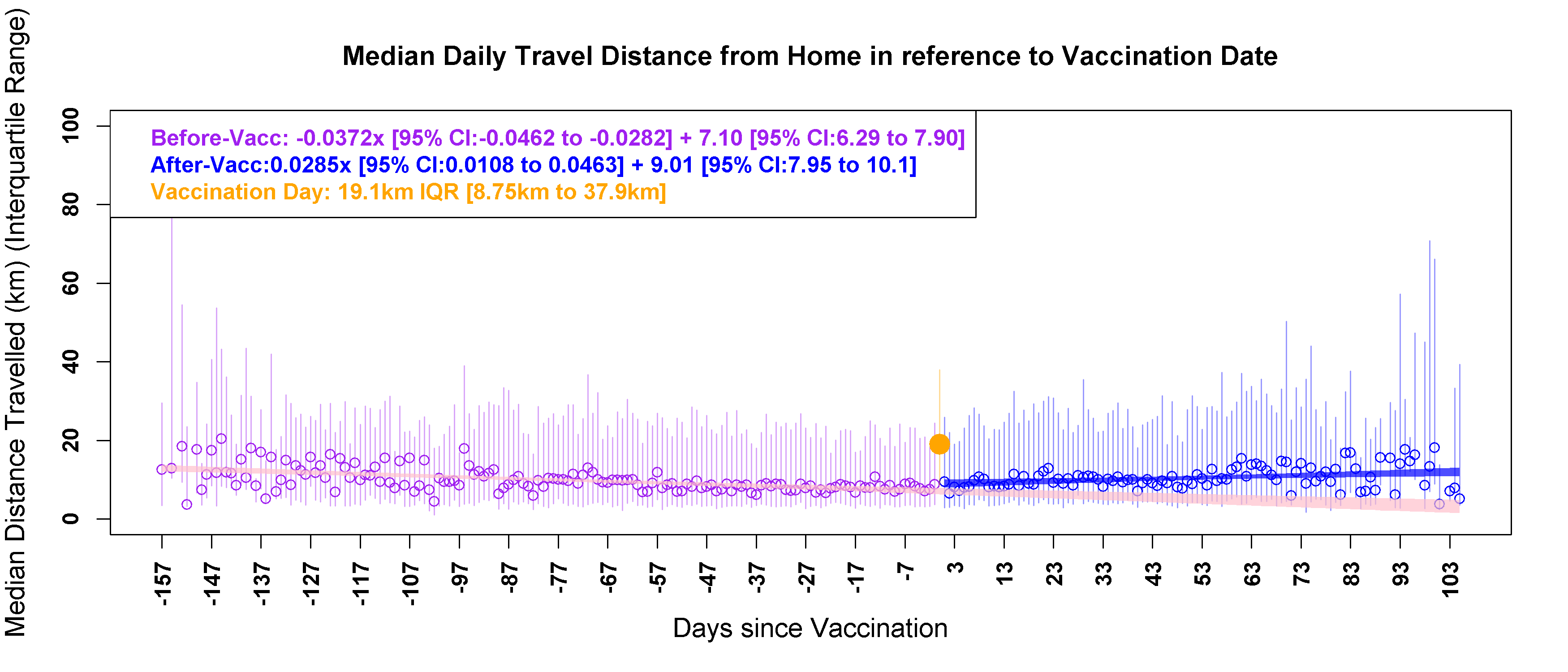

Supplement: Multimedia Appendix 2 [file publichealth_v9i1e38072_app2.png]

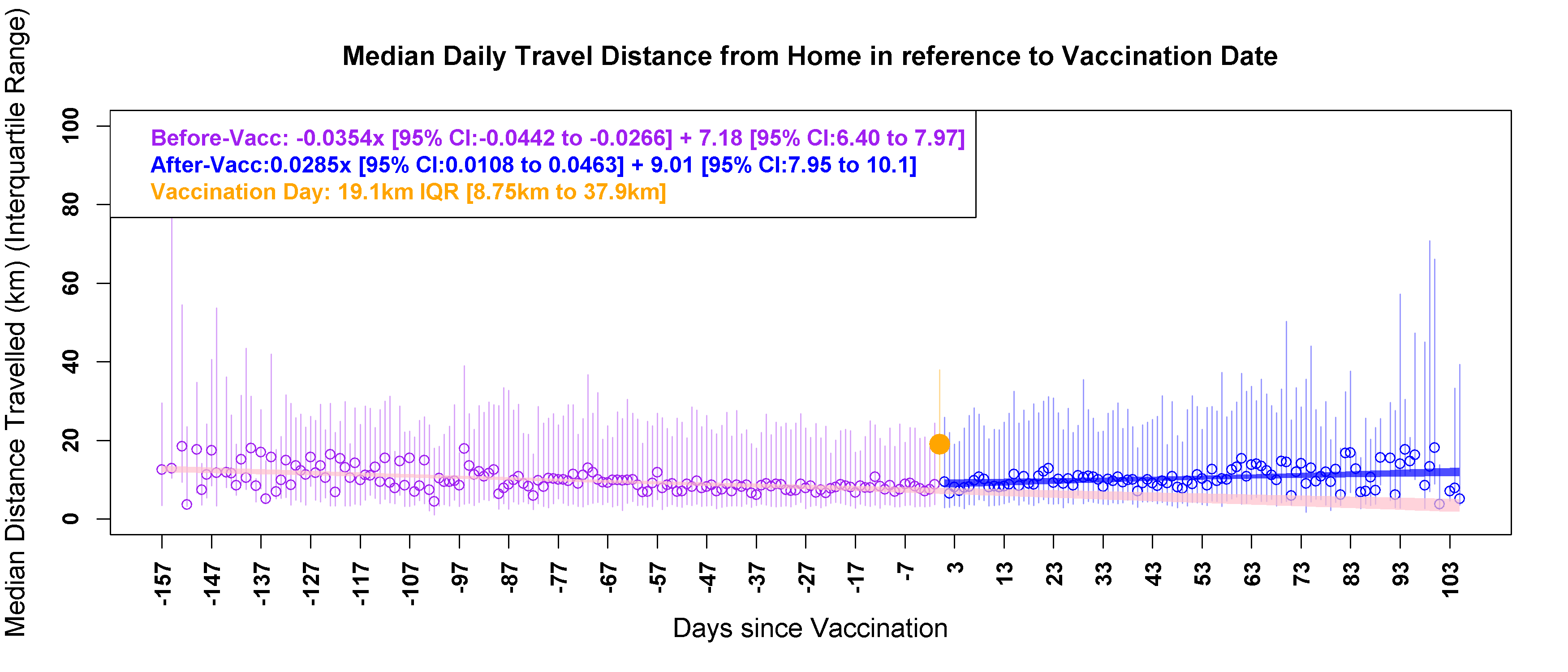

Supplement: Multimedia Appendix 3 [file publichealth_v9i1e38072_app3.png]
